# Regulation of developmentally controlled enhancer activity by extrinsic signals in normal and malignant cells: AP-1 at the centre

**DOI:** 10.3389/freae.2024.1465958

**Published:** 2024-09-16

**Authors:** Alexander Maytum, Nadine Obier, Pierre Cauchy, Constanze Bonifer

**Affiliations:** ^1^ Blood Cell Development Group, Novo Nordisk Foundation Center for Stem Cell Medicine, The Royal Children’s Hospital, Murdoch Children’s Research Institute, Parkville, VIC, Australia; ^2^ College of Medical and Dental Sciences, Institute of Cancer and Genomic Sciences, University of Birmingham, Birmingham, United Kingdom

**Keywords:** signaling pathways, development, activator-protein-1 (AP-1) family of transcription factors, hematopoiesis, enhancers, chromatin priming, VEGF-signaling, RUNX1

## Abstract

The ability of cells to respond to external stimuli is one of the characteristics of life as we know it. Multicellular organisms have developed a huge machinery that interprets the cellular environment and instigates an appropriate cellular response by changing gene expression, metabolism, proliferation state and motility. Decades of research have studied the pathways transmitting the various signals within the cell. However, whilst we know most of the players, we know surprisingly little about the mechanistic details of how extrinsic signals are interpreted and integrated within the genome. In this article we revisit the long-standing debate of whether factors regulating cellular growth (cytokines) act in an instructive or permissive fashion on cell fate decisions. We touch upon this topic by highlighting the paradigm of AP-1 as one of the most important signaling-responsive transcription factor family and summarize our work and that of others to explain what is known about cytokine responsive cis-regulatory elements driving differential gene expression. We propose that cytokines and, by extension, multiple types of external signals are the main drivers of cell differentiation. They act via inducible transcription factors that transmit signaling processes to the genome and are essential for changing gene expression to drive transitions between gene regulatory networks. Importantly, inducible transcription factors cooperate with cell type specific factors within a pre-existing chromatin landscape and integrate multiple signaling pathways at specific enhancer elements, to both maintain and alter cellular identities. We also propose that signaling processes and signaling responsive transcription factors are at the heart of tumor development.

## 1 Introduction

The identity of a cell is defined by a gene regulatory network (GRN) which consists of transcription factors (TFs) binding to their respective target genes including other TF genes, thus forming vast interconnected networks of coordinately and diversely regulated genes (Figure 1A). Such networks can be highly stable as exemplified by cell lines, including pluripotent stem cells, which preserve their identity within a defined culture medium that permits growth without differentiation. Each cell type is defined by the combination of expressed TFs (Valencia and Peter, 2024) but during the development of multicellular organisms, cell types and thus their GRN need to be changed in response to extracellular signals (Peter and Davidson, 2016). Signals can include cell-cell interactions via integrins, growth factor (cytokine) signaling, steroid hormones and metabolic intermediates, involving a multitude of intracellular and surface located molecules that sense the environment, transmit those signals inside the cell via cascades of posttranslational modifications such as phosphorylation and kick off both growth and differentiation. How such a process generates the different cell types and organs is still one of the fundamental questions in developmental biology. It has been proposed that cell fate decisions occur in a stochastic fashion, by cells transiently expressing different gene expression programs compatible with cell differentiation which then reach a threshold leading to a GRN change (Ham et al., 2024; Teles et al., 2013; Pina et al., 2012). It has also been proposed that cytokines act in a permissive fashion which would select cells able to respond to their presence by outgrowing other cell populations (Fairbairn et al., 1993; Robb, 2007). In the last few years evidence has been mounting that cytokines do much more than regulate cellular growth but in fact are intimately involved in regulating cell fate decisions and differentiation dynamics (Sarrazin and Sieweke, 2011; Kull et al., 2022; Rieger et al., 2009) However, the mechanistic details of how cytokines and other signals impact on cell differentiation at the level of the genome are not entirely clear. We know that signaling processes alter the epigenome, via the modification of TFs binding to the cis-regulatory elements (CREs) of genes and the alterations of chromatin components (Mahadevan et al., 1991; Clayton et al., 2000). A multitude of TFs are signaling responsive with respect to their DNA-binding and regulatory activity, causing differential gene activity (Examples are shown in Figure 1B). The question then arises of how such factors, most of which are constitutively expressed, drive changes in cell fate.

**FIGURE 1:**
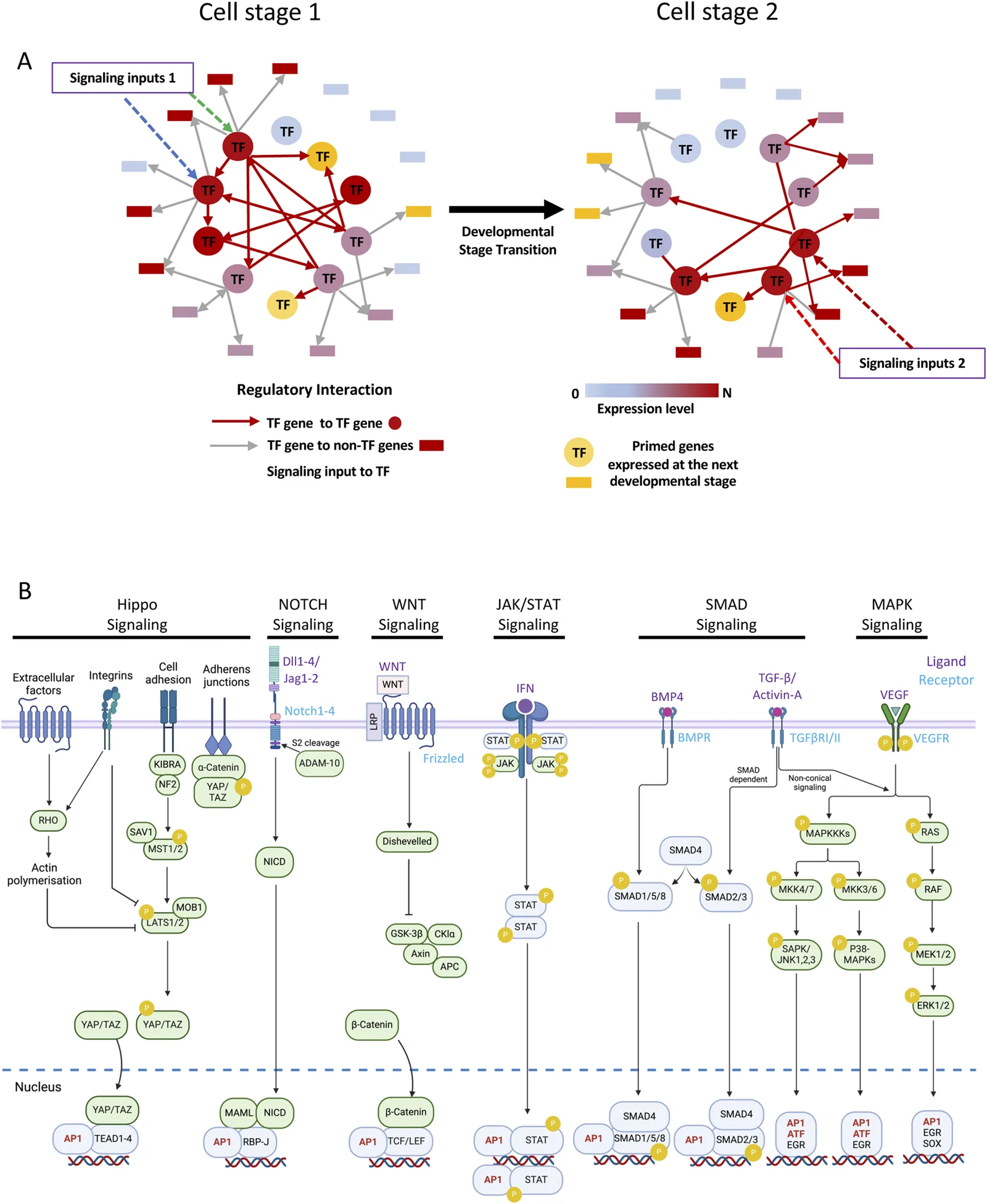
Gene regulatory networks (GRNs) include signaling responsive transcription factors. **(A)** Schematic of a GRN transiting from one cell stage to another in response to signaling as indicated. Arrows (Edges) point from nodes (TF genes) to non-TF genes as indicated below the network diagrams. **(B)** Examples of signaling pathways important for hematopoietic development terminating at AP-1 factors or at signaling-responsive TFs which interact with AP-1 factors. Note that this is a non-exhaustive list. Made in part using BioRender.com.

One of the most important groups of signaling responsive TFs are the FOS/JUN AP-1 family and the ATF family of Leucine Zipper (B-Zip) TFs. FOS and JUN are ubiquitously expressed inducible factors that integrate multiple growth factor signaling via the RAS/Mitogen Activated Kinase (MAPK) pathway (Karin, 1995) (Figure 1B). *JUN* and *FOS* were originally classified as “Immediate Early Response genes” that were activated by serum and multiple other growth stimuli, linking cellular signaling to cell cycle progression (Reviewed in (Ransone and Verma, 1990)). To date, 15 different members belonging to this TF family which function as dimers of the JUN/ATF and the FOS factors have been identified (Mechta-Grigoriou et al., 2001; Bejjani et al., 2019). Figure 2A. RAS signaling activates JUN kinase which phosphorylates JUN and rapidly upregulates *FOS* genes (Waudby et al., 2022). Using their leucine zipper (Figure 2B) JUN factors then pair with FOS factors to bind DNA with high affinity and regulate gene expression (Shaulian and Karin, 2002; Angel et al., 1987). Whilst showing a significant overlap in their set of target genes and function, AP-1 and ATF factors also display unique binding patterns and recognize highly similar but not identical motifs (Fonseca et al., 2019; Obier et al., 2016) (Figure 2C). Consistent with their role in mediating growth factor signaling, AP-1 is required for cell cycle progression, whereby cell cycle genes such as the Cyclin D1 and D2 genes are direct targets and require AP-1 for their activity (Wisdom et al., 1999; Martinez-Soria et al., 2019).

**FIGURE 2:**
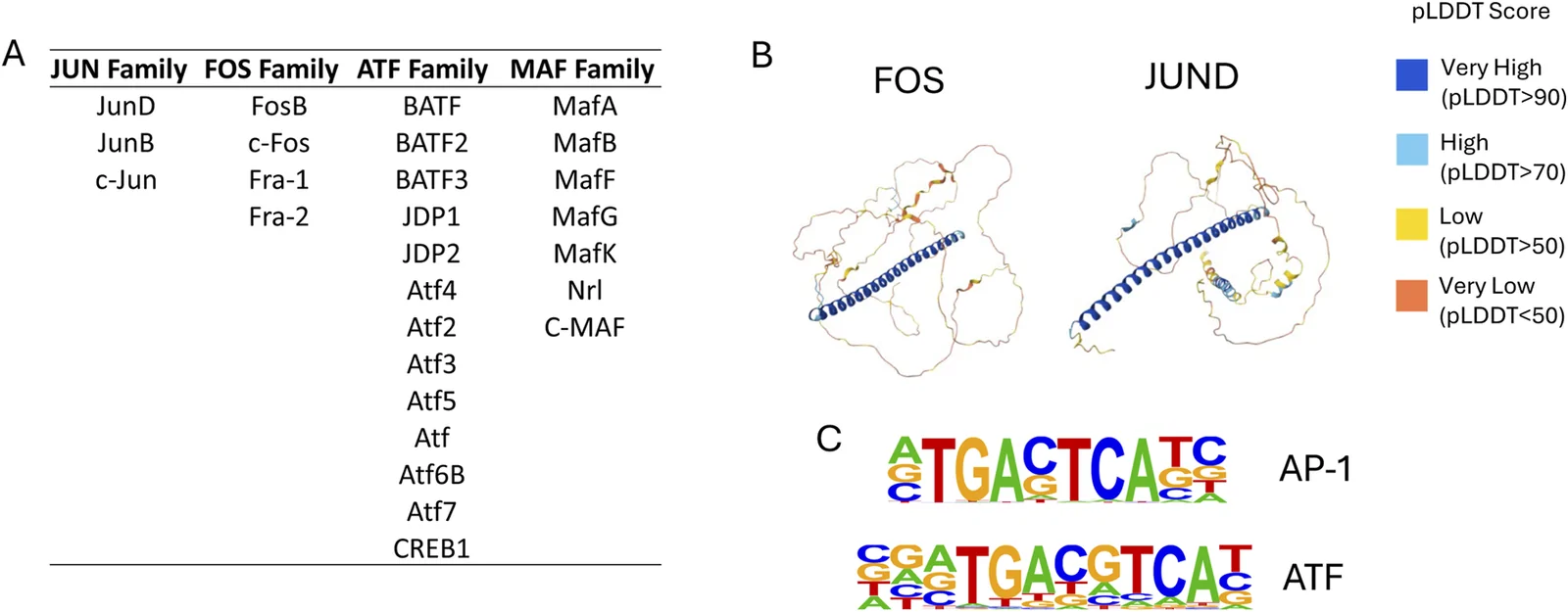
The AP-1 transcription factor family. **(A)** List of JUN, FOS, ATF and MAF TFs. **(B)** Structure of the FOS and JUND proteins with the Leucine Zipper shown in blue (predicted using AlphaFold where the colors stand for the value of the confidence pLDDT score (Jumper et al., 2021). **(C)** DNA sequences recognized by the JUN/FOS dimer and the ATF/FOS dimer.

An important feature of AP-1 factors is their ability to cooperate with other transcriptional regulators, thus influencing tissue-specific gene expression and other signaling-responsive TFs. For example, AP-1 is a major mediator of inflammatory cytokine action, which is inhibited by glucocorticoids. The molecular mechanism by which inhibition occurs involves the interaction of the glucocorticoid receptor (GR) with AP-1, thus blocking its activity (Yang-Yen et al., 1990). Another example of an integration between signaling pathways is the direct interaction and colocalization of MAPK-inducible AP-1 and Ca^2+^-inducible nuclear factor of activated T Cells (NFAT) on a rigidly defined composite DNA-binding motif (Johnson et al., 2004; Brignall et al., 2017). In addition, the interaction between AP-1 and the master regulator of myelopoiesis PU.1 is required for myeloid differentiation (Zhao et al., 2022). JUN is also capable of heterodimerizing with other leucine zipper proteins such as C/EBPα which is essential for macrophage differentiation and the complex binds to a composite hybrid motif with the sequence TGACGCAA at its heart (Hong et al., 2011). Importantly, AP-1 factors also cooperate with members of the chromatin regulatory machinery such as CBP/p300 (Tsai et al., 2008) and the nucleosome remodeling complex SWI/SNF (Vierbuchen et al., 2017; Wolf et al., 2023) to alter chromatin structure.

AP-1 activity differs between family members. AP-1 activity can be suppressed in the absence of signals that normally activate *FOS* and *JUN,* thus ensuring low basal level activity. Although the homeostatic cytokine IL-7 and AP-1 are required for the priming of memory T cells, IL-7 induces JUND but not FOS and JUN (Bevington et al., 2016). This finding is significant because JUND appears to function as an immune suppressor during homeostasis. T cell activation is greater in the absence of JUND (Meixner et al., 2004) and unlike FOS, JUND is expressed in the absence of stimulation in serum-starved cells and is rapidly degraded in response to serum (Pfarr et al., 1994). FRA-1 and FRA-2 also have a dampening effect as they are induced later than FOS and appear to lack transactivation domains (Reddy and Mossman, 2002). The variety of interactions, the complexity and the high level of redundancy between the different family members (Passegue et al., 2002) are the likely reason why mice carrying knock-out alleles of individual family members have highly variable phenotypes, from embryonic lethality (*Jun, JunB)* to a very mild phenotype (*JunD, Fos)* (Mechta-Grigoriou et al., 2001; Chen et al., 2022; Passegue et al., 2004; Jochum et al., 2001; Hilberg et al., 1993; Thepot et al., 2000). To gain insight into the precise mechanism of action of AP-1 proteins in a chromatin context it is therefore required to assess their role in tissue-specific gene regulation. As it turns out, AP-1 pays a crucial role in shaping cellular identities by integrating multiple signaling pathways to coordinate cell proliferation with global CRE activity driving differentiation. The next chapters will summarize our work which used the hematopoietic system as an example to highlight the precise molecular mechanisms of how AP-1 and cytokine signaling influence cell fate decisions and cell growth by regulating the activity of cis-regulatory elements driving the expression of key differentiation genes.

## 2 AP-1 plays important roles at different stages of hematopoietic development

Conditional knock-out of JUNB in hematopoietic stem cells leads to the development of a myeloproliferative disease whereby the target cell of the gene editing event is of the essence for the phenotype, again indicating the cell-type specificity of AP-1 function (Passegue et al., 2004). However, the molecular mechanisms that link this phenotype to gene regulation are still poorly understood. To bypass the problem of redundancy between AP-1 family members, we exploited an embryonic stem cell (ESC) based *in vitro* differentiation system (Obier et al., 2016) which allowed us to perform global multi-omics analyses at multiple differentiation stages of blood cell development from the mesoderm to terminally differentiated cells (Goode et al., 2016). During ESC differentiation, hematopoietic cells arise from the hemangioblast (HB), a mesodermal cell type with the potential to differentiate into vascular smooth muscle (SM), endothelial and hematopoietic cells. Hematopoietic cells directly bud from a hemogenic endothelium (HE) which is an adherent cell type that changes shape to give rise to round and mobile hematopoietic progenitor (HP) cells via an endothelial–hematopoietic transition (EHT)

To study how the abolition of AP-1 activity and thus the response to multiple signaling pathways affected different stages of hematopoiesis, we expressed an inducible version of a dominant-negative FOS peptide (dnFOS) which blocks the binding of all JUN-related proteins to chromatin in ESCs (Olive et al., 1997). We then expressed dnFOS at different stages of ESC derived blood cell development, before and after the EHT and studied cellular phenotypes, JUN/FOS binding to chromatin and global gene expression (Obier et al., 2016). Gene expression was differentially affected at each stage of development. At early stages, FOS and JUN bind to genes involved in blood vessel development, cell adhesion and cell signaling. Inhibition of AP-1 had a surprisingly mild effect at these early stages of hematopoietic specification prior to the EHT but we observed a significant shift in the balance between the endothelial and the hematopoietic program after the EHT with an increase in blood progenitor cells. However, when dnFOS was expressed at the progenitor stage, myelopoiesis was completely blocked with a complete absence of expression of crucial regulators such as PU.1 and C/EBPα, both of which are JUN/FOS targets, defining AP-1 family members as essential for the development of myeloid cells. An interesting finding was that only about 40% of the binding of JUN and FOS bound sites overlapped, highlighting again that individual family members play non-redundant roles in gene regulation and may partner with other TFs.

We also performed digital DNaseI footprinting experiments in sorted HE and HP cells to identify such factors. In the HE, we noticed a high frequency of enrichment and co-localisation of the motif for the HIPPO-signaling mediator TEAD with AP-1 motifs whereby the two motifs showed a defined spatial arrangement which suggested the formation of a complex between the two proteins (Goode et al., 2016). Moreover, we saw a strong co-enrichment of AP-1 with motifs for SMAD transcription factors mediating TGFβ signaling in footprints specific for HE cells (Obier et al., 2016). This co-enrichment was confirmed in the analysis of open chromatin regions in HE cells from mouse embryos where it was shown that inflammatory and TGFβ signaling processes are important for the development of hemogenic endothelial cells (Howell et al., 2021; Li et al., 2014). The nature of co-enriched and co-localizing motifs was completely different in footprints specific for HP cells which were dominated by motifs for the hematopoietic TFs RUNX1, PU.1, ETS and C/EBP, demonstrating that the binding pattern of AP-1 members had completely changed during differentiation. These results put a firm basis under the idea that (i) each cell type displays its own AP-1 binding pattern based on the cooperation with different family members and cell-type specific TFs which (ii) links this factor family to a cell-type specific chromatin landscape. We therefore suggested that signaling processes mediated by AP-1 instruct changes of cell fates by cooperating with pre-existing cell-type specific factors activating the expression of lineage-specific genes instead of acting upon a sub-population of cells and selecting a specific cell population for growth.

### 2.1 The cooperation of AP-1 with TEAD and RUNX1 is required for hematopoietic specification

The question arising from these data was of the functional significance of the colocalization of TEAD and RUNX1 with AP-1. TEAD is a major mediator of HIPPO signaling, which together with its co-factors YAP/TAZ is absolutely essential for the EHT (Goode et al., 2016) where it is required to switch on RUNX1 in response to the onset of blood flow (Lundin et al., 2020). The integration of ChIP data for TEAD and peaks bound by JUN/FOS dimers in FLK1+ cells representing the differentiation stage prior to the HE, showed a significant overlap between the binding sites for the two factors in the HE (Obier et al., 2016; Goode et al., 2016), in particular at vascular genes whose expression was significantly reduced after dnFOS expression. We therefore performed ChIP experiments for TEAD4 before and after dnFOS expression (Figure 3A) and demonstrated that the abrogation of AP-1 binding abolished TEAD binding as well, demonstrating a close collaboration of the two factors in defining gene expression in the hemogenic endothelium and thus integrating several independent signaling pathways (Obier et al., 2016) (a re-analysis of this data is shown in Figure 3B). We have not measured AP-1 binding in the absence of TEAD, but it is likely that the formation of endothelial cells including the HE involves AP-1 dependent TEAD4 *de novo* binding, suggesting a cooperative requirement role for AP-1. Our findings in primary cells adds to the increasing number of studies which described a growth-promoting role of AP-1/TEAD interactions in cancer cell lines demonstrating that this type of signaling integration is wide-spread across multiple tissues (Liu et al., 2016; Zanconato et al., 2015; Koo et al., 2020). However, during hematopoietic specification, AP-1 and HIPPO signaling also play an instructive role as both are required for the establishment (Lundin et al., 2020) and maintenance of the endothelial cell fate, as in the absence of AP-1 the balance is shifted towards hematopoietic cells (Obier et al., 2016).

**FIGURE 3:**
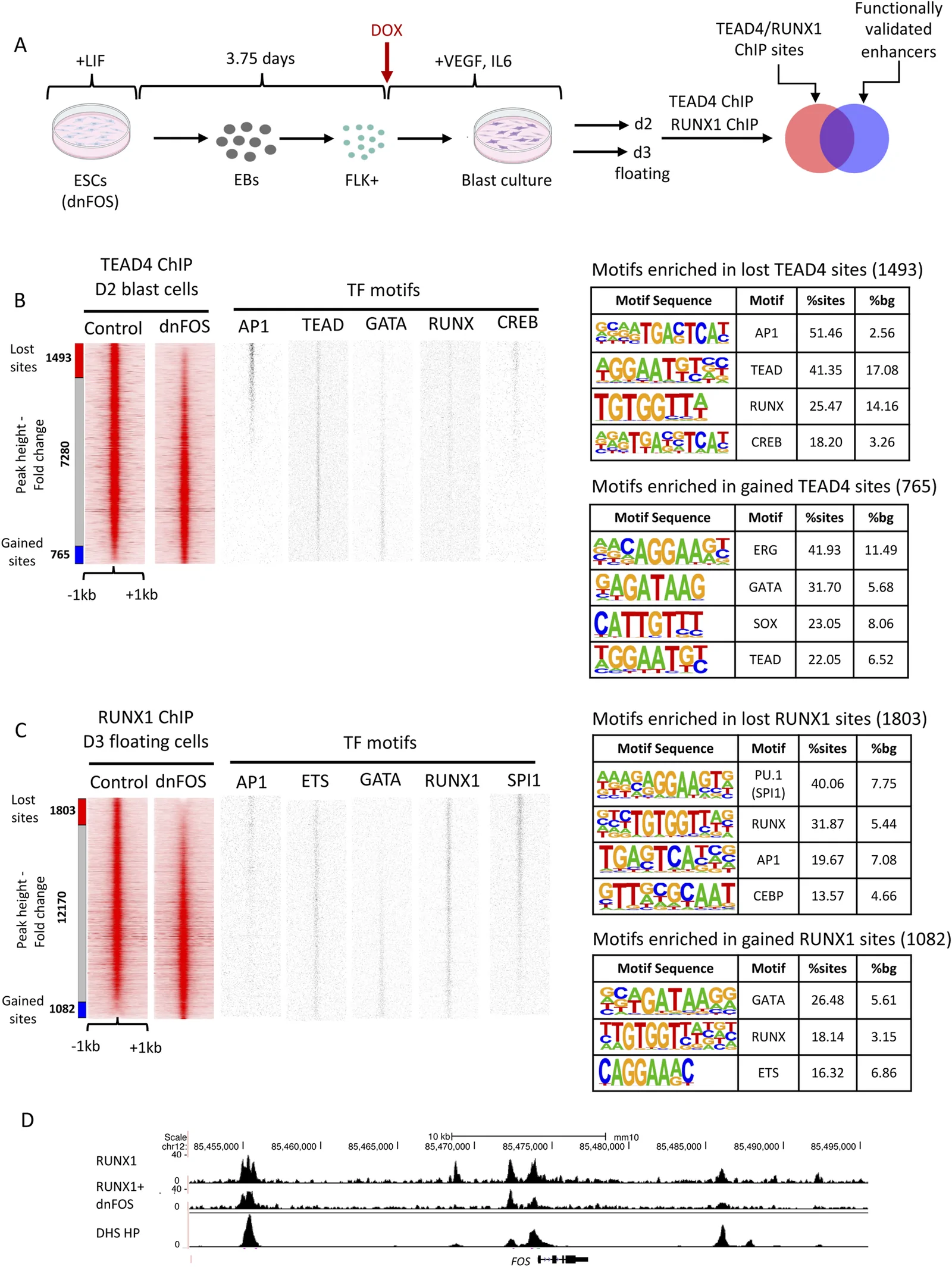
TEAD 4 and RUNX1 binding alter after AP-1 depletion. **(A)** Experimental scheme and cell types analysed. **(B, C)** ChIP experiment measuring TEAD4 binding with D2 FLK1+ blast culture cells **(B)** and RUNX1 binding in day 3 floating progenitor (HP) cells **(C)** from uninduced (-DOX) and induced (+DOX) cells expressing dnFOS. Binding motifs of the indicated TFs are plotted alongside the ChIP data. **(B)** TEAD4 data from (Obier et al., 2016) were re-analysed to permit integration with the new RUNX1 data. Peaks from treated cells were ranked alongside those from untreated cells. 1493 TEAD4 sites were lost (peak height was reduced >2-fold) and 765 sites were gained (peak height increased > 2-fold). **(C)** 1803 RUNX1 sites were lost (peak height was reduced >2-fold) and 1,082 sites were gained (peak height increased > 2-fold). **(D)** UCSC browser screenshot of the *Fos* locus showing RUNX1 binding with and without dnFOS and the pattern of DNaseI hypersensitive sites (DHS) surrounding the gene.

The role of the co-localization of RUNX1 and AP-1 was more difficult to explain. The AP-1 motif was found in both HE1-specific and HP-specific DHSs, but not at dnFOS unresponsive sites, which are dominated by RUNX1 and ETS motifs (Figure 3C, TF motif plots), highlighting a different function of these binding sites in endothelial and hematopoietic cells. RUNX1 is absolutely required for the EHT (Lancrin et al., 2009) and the activation of the hematopoietic gene expression program. Importantly, RUNX1 reorganizes the chromatin landscape by relocating other TFs such as SCL/TAL1, LDB1 and FLI-1 to new binding sites (Lichtinger et al., 2012; Gilmour et al., 2018). It has also been shown previously in T cells that RUNX1 binding can be dependent on the NFAT/AP-1 complex (Johnson et al., 2004). We therefore performed RUNX1 ChIP experiments in the presence and absence of dnFOS in HP cells (Figure 3C). As seen with TEAD, also here the expression of dnFOS leads to a loss of at least 1,800 distal open chromatin regions, but also with a gain of about 1,000 new binding sites (Figure 3C). TF binding motif analysis of lost and gained regions showed a profound loss of binding motifs for RUNX1 and AP-1 (as expected) in addition to those of the myeloid TFs PU.1 and C/EBP family members, providing support for the finding that AP-1 is essential for myelopoiesis (Obier et al., 2016) and adds that the cooperation of AP-1 and RUNX1 is essential for this process. Interestingly, inspection of the genes associated with lost RUNX1 sites (Supplemental dataset 1) revealed numerous transcriptional regulators, including *JUN* and *FOS* themselves suggesting a complex feedback relationship between RUNX1 and the AP-1 factor family (shown for *Fos* in Figure 3D). Gained sites show enrichment of RUNX, GATA and ETS motifs, indicating that RUNX1 moves towards such sites which are characteristic for a stem cell/erythroid fate, again demonstrating that the cooperation of AP-1 with tissue-specific factors determines cellular fates. In summary, our data demonstrate that for the first step in differentiation to occur, such a mechanism does not require to activate a new set of regulators, it only requires to relocate cooperating pre-existing and inducible factors to new cis-regulatory elements and then activate a new set of genes. The question now arose of the nature of the cis-regulatory elements upon which such cooperation takes place.

### 2.2 AP-1 is a ubiquitous component of tissue-specific and signaling responsive enhancer elements

The developmental control of gene expression critically depends on enhancer elements which interact with promoters to activate gene expression and define the dynamics of expression of a given gene in a specific regulatory context, and the hematopoietic system is not exception (Field and Adelman, 2020; Mulet-Lazaro and Delwel, 2023). Multiple surrogate assays based of chromatin structure and histone modifications have been used to measure the number of enhancer elements in the genome. However, the definition of an enhancer element is functional, i.e., the ability to stimulate the transcriptional activity of a promoter (reviewed in (Field and Adelman, 2020; Mulet-Lazaro and Delwel, 2023)). In order to functionally identify which CREs had the ability to stimulate reporter gene expression in a chromatin context during hematopoietic specification, we developed a high-throughput method based on isolated ATAC-Seq fragments that identified thousands of differentially active CREs able to stimulate a minimal promoter integrated within a safe harbor site within the genome of mESCs (Edginton-White et al., 2023). This system enables to measure enhancer and promoter activity at each stage of differentiation into blood and identified thousands of differentially active CREs. Enhancer elements could also be tested individually to examine the role of specific TF binding motifs. Once defined as functional enhancers, we could then test (i) at which developmental stage such elements were visible as an open chromatin site, (ii) at which stage they develop enhancer activity and (iii) whether such elements are responsive to extrinsic signals and when/how (Edginton-White et al., 2023; Maytum et al., 2024).

Several results were noteworthy: (i) After we filtered out repeat and promoter elements, the majority of distal open chromatin regions showed stimulatory activity in our assay; (ii) the presence of open chromatin did not always indicate that this element was capable to reading out in the enhancer assay at this developmental stage which we defined as a primed state, and (iii) thousands of open chromatin regions were dependent on extrinsic signals which we showed by omitting specific growth factors from the differentiation cultures. Open chromatin was measured in sorted cells making sure that differences in cell type composition were accounted for (Figure 4). We measured the effects of 4 different cytokines: BMP4, which is important to pattern mesoderm, vascular endothelial growth factor (VEGF) which is required to generate endothelial cells, including the hemogenic endothelium (Ottersbach, 2019; Maytum et al., 2023) and Interleukin 3 and 6 both of which are required for the growth of multiple blood lineages (Broughton et al., 2012; Hirayama and Ogawa, 1996) (Figure 4A). Moreover, (iv) when we constructed GRNs from this data, we observed that a subset of connections within the GRN of one developmental stage anticipated the next state, with specific enhancers being organized in open chromatin prior to the onset of gene expression at the next stage, demonstrating that chromatin opening follows a defined trajectory. Some of these sites were also signaling responsive, suggesting a role of cytokines in chromatin priming (Maytum et al., 2024). Finally, (v) co-localization analysis showed that AP-1 motifs were ubiquitously present, sitting next to different cell-type specific factors at each differentiation stage, with AP-1-TEAD pairs being prominent at the endothelial stage and AP-1 shifting its alliance to hematopoietic TFs as seen before. The ChIP assays confirmed this notion. Figure 5 illustrates that the vast majority of dnFOS responsive TEAD4 and RUNX1 binding sites are localized in functionally active enhancers (Figure 5A), with AP-1-TEAD pairs being prominent at the endothelial stage and AP-1 shifting its alliance to RUNX1 at the hematopoietic progenitor stage (Figure 5B).

**FIGURE 4:**
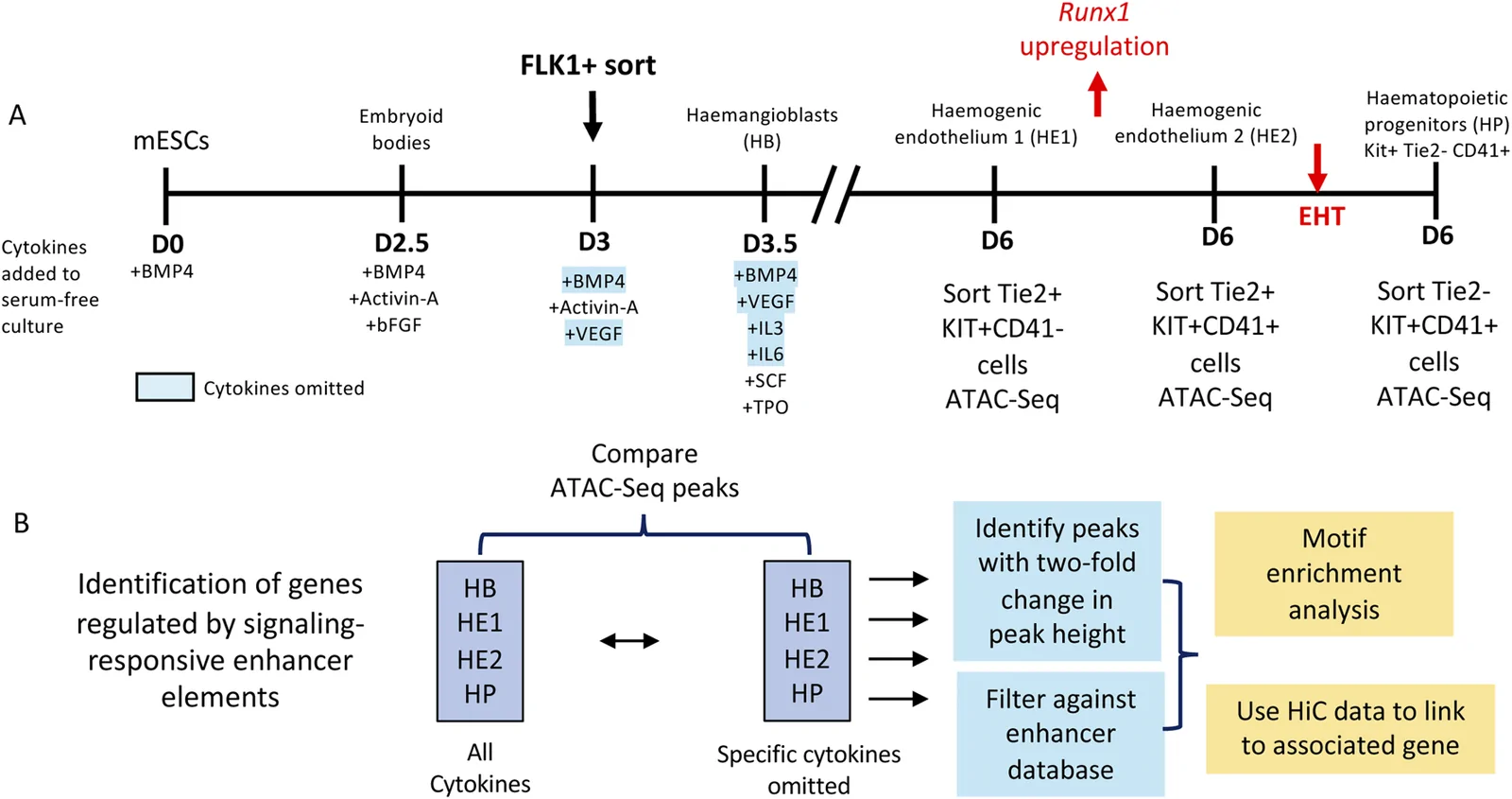
Identification of signaling responsive enhancer elements. **(A)** Scheme of the differentiation system, including cell sorting parameters and **(B)** the workflow to identify signaling responsive enhancers and their associated genes.

**FIGURE 5:**
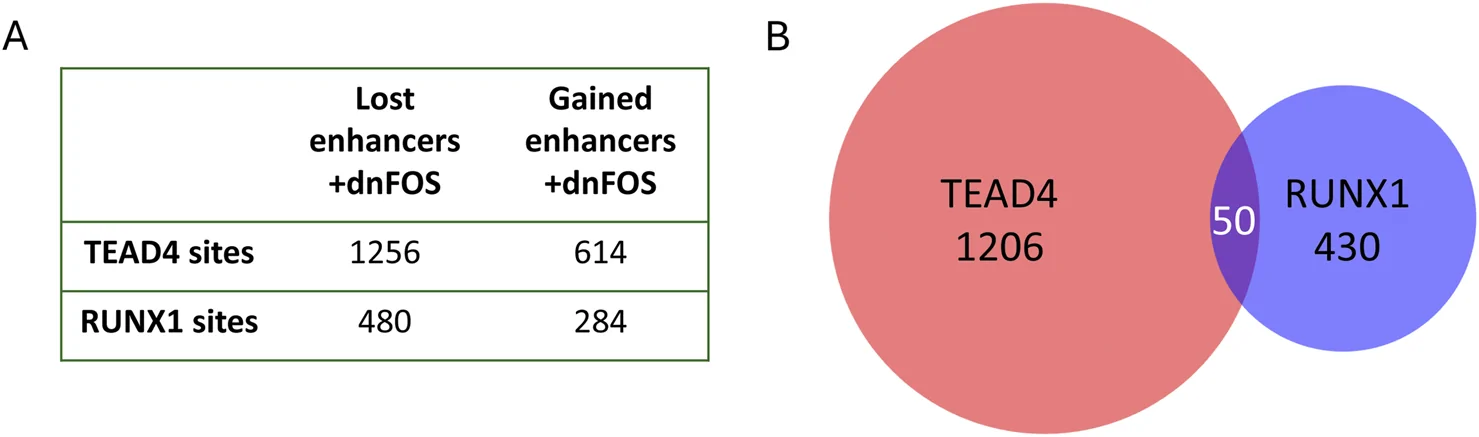
AP-1 binds to different enhancers in the HE and in HP cells. **(A)** Number of dnFOS-responsive TEAD4 and RUNX1 sites which are enhancers. Sites bound by FOS in the HE were intersected with our enhancer database and then were intersected with enhancers active in HE and HP. **(B)** Intersect of TEAD4 and RUNX1 bound enhancers in HE and HP cells.

To understand the molecular basis of cytokine responsiveness of enhancer elements we examined one signaling pathway (VEGF signaling) in more detail (Edginton-White et al., 2023). Strikingly, more that 8,000 enhancers were responsive to the presence or absence of VEGF across all differentiation stages, i.e., showed an at least two-fold change in chromatin accessibility. A careful analysis of the TF binding motifs underlying responsiveness showed that the presence of VEGF was associated with the activation of CREs carrying AP-1 and TEAD motifs, in concordance with the finding that VEGF signals via AP-1 (Kellaway et al., 2024; Jia et al., 2016). The absence of VEGF was associated with the enrichment of RUNX1 motifs, suggesting that VEGF suppresses the activation of such enhancers. Indeed, timed withdrawal experiments demonstrated that hematopoietic development was strongly suppressed by VEGF. Moreover, the presence of VEGF is absolutely required for the establishment of non-hemogenic endothelium but then the maintained signaling input from VEGF suppressed the activation of enhancer elements important for the expression of *Runx1*, thus blocking the full execution of the EHT which requires RUNX1 upregulation. In addition, the removal of VEGF upregulated a repressor of NOTCH-signaling which needs to be down-regulated for the EHT to occur. To study the interaction of VEGF signaling responsive AP-1 factors and TEAD factors we interrogated one enhancer (for the *Galnt1* gene) which we identified as being active in the HE and repressed after the EHT which contained both AP-1 and TEAD binding motifs and which we confirmed by ChIP-Seq was bound by TEAD4, FOS, JUN in the HE and then RUNX1 in the HP (Edginton-White et al., 2023). It turned out that its activity was regulated by a composite AP-1/TEAD element with a 7 base pair space bioinformatically identified in (Obier et al., 2016) which overlapped with a RUNX1 site, thus suggesting that the balance between the three factors determined whether an element was activated after the EHT or not.

To examine this result at the global level we integrated our dnFOS data with the enhancer data and found approximately one thousand enhancers bound by FOS. Only about 50% of these elements were still active in HP cells indicating that half of FOS bound enhancer sites were lost, again showing the cell-type specificity of AP-1 binding. Very few enhancers bound by FOS in the HE were only active in HP cells. To test whether dnFOS responsive TEAD and RUNX1 binding sites were also responsive to VEGF signaling, we integrated these data with ATAC-data from HE and HP cells formed in the presence and absence of VEGF. We found that 44% of all dnFOS responsive TEAD4 binding sites and and 52% of dnFOS-responsive RUNX1 binding sites were also VEGF responsive, confirming that the cooperation between AP-1 and these factors is driven by signaling. The conclusion from these experiments is therefore that the presence or absence of cytokines has a profound and cell-type specific influence on the chromatin landscape and on cell differentiation via the differential activity of enhancer elements.

### 2.3 The AP-1 and RUNX1 axis drives cell cycle progression and is essential for acute myeloid leukemia development

Both the *JUN* and the *FOS* genes were originally isolated from oncogenic retroviruses suggesting that their mis-expression could transform cells (Ransone and Verma, 1990). The fact that cell cycle progression and AP-1 activity are closely linked has long been recognized as an essential part of the cellular transformation process (Shaulian and Karin, 2002; Angel and Karin, 1991) as AP-1 inhibition blocks oncogenesis in multiple contexts (Olive et al., 1997; Tichelaar et al., 2010). This is also true for Acute Myeloid Leukaemia (AML). The analysis of the GRNs maintaining mutation-specific AML sub-types revealed that AP-1 binding sites are a prominent node in the GRN of all studied sub-types. Moreover, employing the dnFOS peptide, we could show that blocking AP-1 DNA binding activity blocked the proliferation of several AML sub-types *in vitro* and *in vivo* (Martinez-Soria et al., 2019; Kellaway et al., 2024; Assi et al., 2019; Adamo et al., 2023; Coleman et al., 2023). The same was true for RUNX1. Inhibition of the DNA binding activity of this TF using a small molecule inhibitor (Illendula et al., 2016) or down-regulating its expression blocked the growth of multiple AML sub-types as well (Adamo et al., 2023; Coleman et al., 2023; Ben-Ami et al., 2013; Wesely et al., 2020). For two AML sub-types, the t (8; 21) translocation which expresses an aberrant RUNX1 fusion protein, RUNX1-ETO and the FLT3-ITD which expresses a constitutively active receptor for the cytokine FLT3-ligand, we were able to gain insights into the molecular mechanism of RUNX1 action, the signals activating AP-1 and its genomic targets involved in driving cell proliferation. As it turned out, the signals were highly heterogeneous. The molecular basis of driving proliferation was not.

In recent years, several inhibitors of FLT3 signaling were developed that provided temporary clinical benefit for patients, but most of them eventually relapsed (Perl et al., 2022). To gain insight into why this was the case, we generated GRNs of cells from patients before FLT3 inhibitor (FLT3i) treatment and after relapse (Coleman et al., 2024). We also studied a patient that was unresponsive to the drug. The comparison of the AML-specific GRN before and after relapse showed that the biggest changes were seen in the AP-1 and the RUNX1 nodes. AP-1 mediated connections were upregulated, whilst RUNX1 connections were down-regulated. The unresponsive patient showed no or little change in their GRN. To test, how FLT3i affected AP-1 binding in responsive cells that stopped proliferating after inhibitor treatment, we performed ChIP assays which demonstrated that both AP-1 and RUNX1 binding to chromatin was down-regulated, demonstrating that AP-1 was the main mediator of FLT3 signaling with the expression of cell cycle genes being strongly affected. Interestingly, the down-regulation of binding occurred at sites with co-localizing AP-1/RUNX1 motifs and as seen during hematopoietic specification (Figure 3), experiments using dnFOS showed that RUNX1 binding at these sites was indeed dependent on AP-1 binding. Moreover, the small molecule inhibition of RUNX1 also led to a profound cell cycle block (Coleman et al., 2023). The explanation as to why RUNX1 connections were lost after relapse was somewhat unexpected. FLT3i treatment and the cell cycle block led to a strong upregulation of multiple signaling genes, such as *KIT*, all of which are targets of RUNX1 which primed the cells for bypassing inhibition of the FLT3 signal by using signals from other cytokines. Indeed, the addition of cytokines such as IL-3 immediately restored growth and RUNX1 binding. In relapse patients, RUNX1 was still required for growth, but its GRN was rewired towards a lesser dependence on RUNX1.

The precise molecular mechanism by which the AP-1/RUNX1 axis drives growth in t(8;21) cells was completely different (Kellaway et al., 2024). Here, the driver oncogene, RUNX1-ETO directly influences the cell cycle by counteracting the role of normal RUNX1 (Martinez-Soria et al., 2019; Nafria et al., 2020). However, the cells strike a fine balance because wild-type allele of RUNX1 is still present and, as in other AML types, is required for growth (Ben-Ami et al., 2013). To develop rapidly growing blast cells expressing RUNX1-ETO, cells therefore acquire activating mutations in signaling pathways such as those generating a constitutively active RAS or KIT (Krauth et al., 2014). However, this type of bypass only operates in blast cells, as it was shown that the activation of such genes in HSCs on their own leads to stem cell exhaustion (Di Genua et al., 2019). In leukemic stem cells (LSCs) which are mostly quiescent, the IL-5 and VEGF pathways are activated which both signal to AP-1 and in HSCs still are organized in a chromatin structure that is permissive for activation (Kellaway et al., 2024). Once these pathways are active, LSCs start to grow and differentiate into leukemic blast cells. dnFOS expression leads to a loss of RUNX1 binding in chromatin, again showing that proliferation is regulated by the RUNX1/AP-1 axis. The combination of signaling mediated AP-1 activity and AP-1-dependent RUNX1 binding therefore generates a feed-forward loop that kick-starts leukemic growth.

### 2.4 AP-1 links signaling to nucleosome displacement and enhancer-promoter loop formation

The examples described above give a brief glimpse into the breath-taking complexity of mechanisms and pathways of how normal and malignant cells use AP-1 to integrate multiple extrinsic signals in a chromatin environment to control cell fate via the activity of thousands of enhancer elements and cell proliferation via the direct regulation of cell cycle and signaling genes. JUN/FOS are relatively small proteins with a simple structure (Figure 2B). The only discernable domain is the leucine-zipper domain, whilst the rest of the protein and its transactivation domain are intrinsically disordered (Campbell and Lumb, 2002) (Figure 2B). How do they do it?

The answer, as already alluded to above, lies in the intrinsic ability of these proteins to use their flexible trans-activation domain to interact with multiple other TFs. Such interactions can be guided by DNA on composite binding sites, as exemplified by the AP-1/NFAT complex (Johnson et al., 2004; Chen et al., 1998), by interdependent binding of juxtaposed individual binding sites as seen with TEAD, or by protein-protein interactions as exemplified by PU.1 (Zhao et al., 2022) and the GR (Yang-Yen et al., 1990). However, it is the interaction of AP-1 with co-factors that is the key to linking extrinsic signals to the binding of these factors to enhancer activity. Seminal work by Gordon Hager studied the interaction of AP-1 with the GR and provided a first insight into how the binding of AP-1 facilitates the binding of other factors to chromatin. The GR is unable to bind to nucleosomal DNA by itself, requiring help from AP-1 factors recruiting SWI/SNF chromatin remodeling complexes. AP1 may even act in a transient fashion, a process defined as “assisted loading” (Biddie et al., 2011; Voss et al., 2011). As it turns out, the majority of AP-1/GR bound sites are occupied by nucleosomes which would support a model of enforced cooperativity in the binding of these two factors (He et al., 2013). It was also shown that AP-1 is a major interactor of the SWI/SNF complex and is required to recruit it to chromatin (Wolf et al., 2023). An elegant study by Vierbuchen et al. (2017) used fibroblasts from hybrid mouse strains to home in on regions where the cooperative binding of AP-1 with other factors is abolished by a sequence variation in individual alleles. Again, at these elements AP-1 recruits the BAF complex which facilitates tissue-specific TF binding and has been shown to be required for enhancer function (Alver et al., 2017). AP-1 is not only required for enhancer activity but also plays an important role in regulating signaling-dependent changes the 3D nuclear structure (Phanstiel et al., 2017). Given the sheer number of proper enhancers with AP-1 binding motifs in the genome, it does not come as a surprise that overexpression of JUN is incompatible with maintaining pluripotency and presents a barrier to cellular reprogramming as the fine balance of changes in TF complexes is disturbed and inappropriate enhancers are activated (Liu et al., 2015). In summary, these and our studies show that (i) enhancer activity regulated by signaling-dependent AP-1 binding is wide-spread and (ii) that signaling dependent AP-1 activity is one of the major driving forces in defining and changing cellular identities via chromatin reorganization and differential gene expression.

As described above, chronic AP-1 activation is a hallmark of AML and other cancers and drives malignant growth. A final aspect of AP-1 mediated transcriptional mechanisms is how its activity is being turned off. FOS expression is mainly regulated transcriptionally, via a serum-response element binding the TF ELK (Cavigelli et al., 1995) and by responding to MAPK signaling with an increase in Polymerase II initiation (burst) frequency (Senecal et al., 2014). JUN Kinase phosphorylates 4 sites in the TA-domain in JUN, but with different kinetics, whereby the phosphorylation of the first two sites recruits co-activators and subsequent phosphorylation shifts the balance towards co-repressor recruitment (Waudby et al., 2022). A similar mechanism has also been observed with ELK (Mylona et al., 2016) thus turning the activation of immediate-early genes and their products into a carefully orchestrated dance between activation and repression, thus fine-tuning gene expression control in response to the cellular environment. In this respect it is interesting to note that the cell-cycle repressor p57/KIP forms a complex with JUN that interferes with co-repressor recruitment, thus leading to an increase in AP-1 transcriptional stimulatory activity at the end of each cell cycle, thus preparing for the next one (Kullmann et al., 2021).

### 2.5 Signaling modifies chromatin and other transcription factors

It should be noted that signaling does not just regulate inducible TFs. The list of TFs that are subject to post-transcriptional modification continues to grow, whereby such modifications often influence protein stability and co-factor recruitment. RUNX1 phosphorylation at multiple sites in response to external stimuli is a prime example (Thomson et al., 1999; Berger, 2010). Another example is GATA1 whose activity and protein stability are altered by acetylation and phosphorylation in response to EPO signaling (Boyes et al., 1998; Hernandez-Hernandez et al., 2006; Zhao et al., 2006). Acetylation influences the interaction of GATA1 with the Bromo-domain protein BRD3 which stabilizes the binding of GATA1 protein complexes in chromatin (Lamonica et al., 2011; Roe et al., 2015). Moreover, signaling molecules can also modify chromatin (histone and non-histone proteins) directly, thus regulating the expression of signaling responsive genes including *JUN* and *FOS* (Mahadevan et al., 1991; Pokholok et al., 2006; Pogna et al., 2010). The genomic response to cellular signaling therefore involves the formation of interacting, dynamic regulatory complexes in chromatin that facilitate gene expression and drive differentiation and proliferation, again demonstrating the direct impact of the signaling environment on cellular identity.

## 3 Conclusion and perspective

The studies described above show in fine detail that signaling processes mediated by cytokines are truly instructive with regards to the determination of cell fates. Our experiments examining the influence of cytokines on enhancer activity such as IL-3 or BMP4 suggest that this notion does not only hold true for MAPK signaling, but also for other signaling processes operating via inducible TFs such as STAT and SMAD factors. The sheer number of signaling responsive enhancer elements which goes into the thousands suggests that cytokine signaling is a main driver of differentiation and acts on the expression of genes essential for differentiation. The prime example for this notion is *Runx1* whose expression is crucial for the endothelial hematopoietic transition but is blocked in the presence of VEGF (Edginton-White et al., 2023). Multiple genes required for hematopoiesis such as *Spi1* (PU.1) and *Cebpa* and *Runx1* itself are RUNX1 targets, its absence therefore blocks the shift from an endothelial to a hematopoietic GRN in its tracks. The same is also true for human cells. The majority of CREs that have been assigned enhancer activity in mouse cells, are also found in hematopoietic cells differentiated from human induced pluripotent stem cells (A.Maytum, unpublished observation). Moreover, akin to what is seen in the mouse, the continuous presence of VEGF also represses *RUNX1* upregulation in a human serum-free differentiation system and affects the development of HSCs In this study withdrawal of VEGF after endothelial cell formation but prior to the EHT resulted in an increased commitment towards HE and a significant increase in the number of HSCs formed. Furthermore, VEGF withdrawal dramatically increased the transplant efficiency of human iPSC-derived HSCs in an immunocompromised mouse xenotransplantation model (Ng et al., 2024)

Cytokine signaling also affects chromatin priming (Maytum et al., 2024; Bevington et al., 2020). An example for this idea is the effect of IL-3 which signals via the JAK/STAT pathway. Its absence *in vivo* leads to a delay in HSC development. It has been demonstrated to be a survival factor that regulates proliferation of HSCs in the developing mouse embryo prior to the stage at which HSCs are normally detected (Robin et al., 2006). *In vitro*, IL-3 treatment profoundly influences the enhancer landscape in the hemogenic endothelium, with STAT3 motifs being lost when it is absent which is consistent with a role of IL-3 in priming hematopoietic development (Edginton-White et al., 2023). One of the enhancers in the common IL3 receptor beta chain gene (*Csf2rb)* is indeed primed prior to the onset of its expression and responds to cytokine treatment (Maytum et al., 2024). A similar mechanism is seen in T cell development where chromatin priming by IL-2 is established prior to the binding of lineage determining TFs (Bevington et al., 2020). An interesting result from this study is the finding that the maintenance of T-cell memory from such cells, which requires the retention of an open chromatin state after an initial induction to facilitate a rapid recall response, is dependent on low level signaling from IL-7. This low-level stimulation maintains primed AP-1/RUNX1 chromatin sites and prevents them from decaying, suggesting that AP-1 mediated signaling does not just alter, but also maintain cell fates. It therefore does not come as a surprise that deregulated AP-1 activity is also associating with ageing (Patrick et al., 2024).

The consequences of our conclusions are wide-reaching. Figure 6 summarizes what we know about the different levels at which AP-1 factors impact on gene expression. It is likely that a similar complexity is found with other signaling pathways as well. Cells will show a different gene expression profile *in vivo* depending on where they are located and which cells they interact with, and recent single cell gene expression data confirm this notion (Pan et al., 2024; Lohoff et al., 2022). The integration of different signaling pathways allows the generation of molecular gradients fine-tuning differentiation and generating polarity. This process has been seen in *Drosophila* embryogenesis for a long time and has also been noted in vertebrates (see for example, (Yanagida et al., 2020)). The balance between different AP-1 family members, i.e., the “AP-1 code” can determine how cells respond to external signals as seen in (Comandante-Lou et al., 2022). Keeping cells in culture and maintaining their phenotype requires balancing proliferation and differentiation which differs from the dynamic situation seen *in vivo*. The same holds true for in cells generated from *in vitro* differentiation systems, such as pluripotent stem cells. Such cell can only be identical to *in vivo* generated cells once the precise order of signaling dependent chromatin gene activation has been recapitulated and the correct order and time point of signals driving enhancer activation has been achieved. Single cell and spatial transcriptomics technologies that enable investigation of multiple features in individual cells, will certainly help to achieve this goal.

**FIGURE 6:**
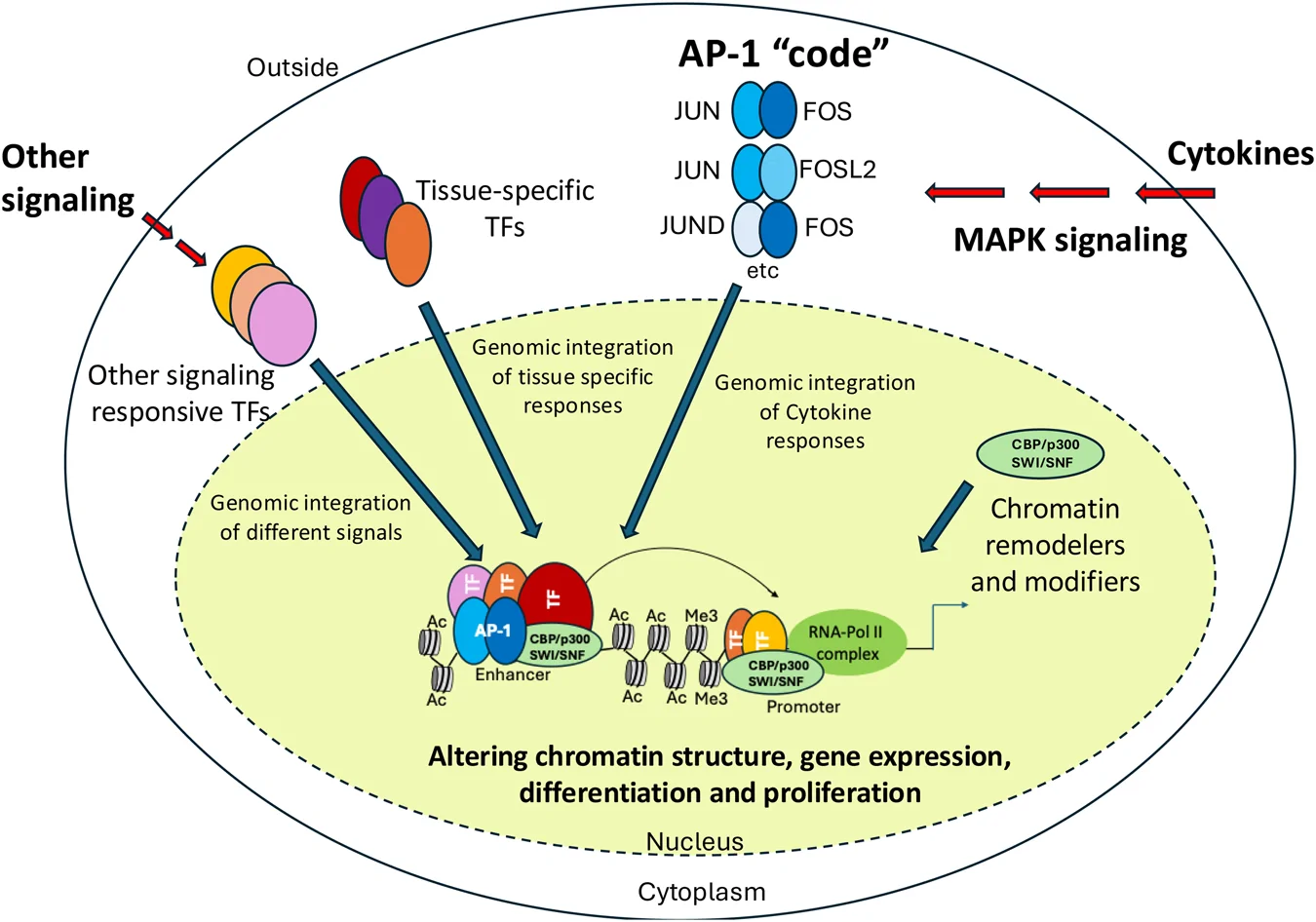
Impact of AP-1 on gene expression. Model explaining how different combinations of AP-1 family members cooperate with chromatin remodellers/modifiers as well as tissue-specific and other signaling-dependent TFs to up-regulate gene expression in response to external signals and developmental cues. AP-1 dimers, chromatin modifiers/remodelers and other TF classes are indicated by different shapes and colors. Histone tail modifications mediated by recruited chromatin modifiers are indicated.

Finally, with very few exceptions, most efforts to stop aberrant cancer growth by targeting individual signaling pathways have failed. Whilst cytokine receptors act only on specific cell types, the cellular systems regulating growth are hugely redundant. Our data show that signaling pathways can be easily rewired and some cytokines can substitute for others. Therefore, we need to consider approaches that targets malignant cells at the source of their aberrant behavior, i.e., the (epi)genome. Targeting AP-1 activity has for decades been recognized as a promising cancer therapeutics, but without much success (Song et al., 2023). However, in the advent of being able to target transcription factors for degradation and combined with the development of refined delivery methods, modulating tissue-specific AP-1 activity would arguably be possible.

## 4 Methods

### 4.1 Cell culture and RUNX1 ChIP

ESCs expressing an inducible dominant negative FOS (dnFOS), generated in Obier et al., 2016 (Obier et al., 2016), were differentiated for 2d to floating hematopoietic progenitors with or without Doxycycline induction and materials were processed exactly as described in (Obier et al., 2016). Cells were crosslinked with Di (N-succinimidyl) glutarate (DSG) and formaldehyde. ChIP on double-crosslinked chromatin was performed using an anti-RUNX1 antibody (Abcam #23980). Sequencing libraries were prepared using the KAPA Hyper Prep Kit as exactly described in (Obier et al., 2016).

### 4.2 ChIP-Seq analysis

TEAD4 dnFOS ChIP-Seq data from (Obier et al., 2016) was downloaded from GSE79320. RUNX1 dnFOS ChIP data was generated as described above. For TEAD4 and RUNX1 dnFOS data raw sequencing reads were trimmed using Trimmomatic version 0.39 (Bolger et al., 2014) to remove low quality sequences and adaptors. Reads were then aligned to the mouse 10 mm genome using Bowtie2 version 2.3.5. (Langmead and Salzberg, 2012). using the parameters–very-sensitive-local. PCR duplicates were removed using Picard tools version 2.20.2 using the MarkDuplicates function (https://broadinstitute.github.io/picard). Peaks were called using MACS2 using the options -q 0.05 –keep-dup all -B--trackline. Peaks were then filtered against the 10 mm blacklist and 10 mm repeat list. For each comparison a peak union was then formed and peaks were extended 200 base pairs around the peak summit and then merged using the merge function in bedtools version 2.27.1 (Quinlan and Hall, 2010) AnnotatePeaks.pl from Homer version 4.11 was used to calculate the average tag count in peak regions with the options -size 200 -bedgraph using the bedgraphs returned from MACS2. Peaks were then filtered against DNase1 data from (Goode et al., 2016). Tag counts were then normalized in R version 4.4.1 as tag-count per million and a peak was taken as being differentially accessible if it had a 2-fold increase or decrease between the two samples before and after Dox induction. Tag-density plots were created by first ranking peaks by fold-difference. The read density was determined using the annotatePeaks.pl function in Homer version 4.11 (Heinz et al., 2010) using the options -size 2000 -hist 10 -ghist -bedgraph. The output was then plotted as a heatmap using Java TreeView version 1.1.6r4 (Saldanha, 2004). Fold-change analysis centred on RUNX1 peaks was performed as previously described (Obier et al., 2016)

Homer was used to perform a *de-novo* motif search on differentially bound peak sets using the findMotifGenome.pl function. The sites of enriched motifs were determined using the annotatePeaks.pl function in Homer using the options -size 2,000 -hist 10 -ghist -m and plotted using Java TreeView.

Sites which were differentially bound by TEAD4 or RUNX1 before and after dnFOS induction were intersected with functionally validated enhancers from to (Edginton-White et al., 2023) to generate lists of enhancers bound by TEAD4 and RUNX1. TEAD4, RUNX1 and FOS differentially bound sites were also intersected with VEGF responsive enhancers using bedtools intersect. Venn diagrams were made using BioVenn (Hulsen et al., 2008)

## Data Availability

The datasets presented in this study can be found in online repositories. The name of the repository is Gene Expression Omnibus https://www.ncbi.nlm.nih.gov/geo/ Accession numbers are: GSE79323. RUNX1 binding data are deposited under GSE274630.
